# Artificial intelligence-driven design of fuel mixtures

**DOI:** 10.1038/s42004-022-00722-3

**Published:** 2022-09-16

**Authors:** Nursulu Kuzhagaliyeva, Samuel Horváth, John Williams, Andre Nicolle, S. Mani Sarathy

**Affiliations:** 1 Clean Combustion Research Center (CCRC), Physical Sciences and Engineering Division, King Abdullah University of Science and Technology (KAUST), Thuwal, 23955-6900 Saudi Arabia; 2 Visual Computing Center (VCC), Computer, Electrical and Mathematical Sciences & Engineering Division, KAUST, Thuwal, 23955-6900 Saudi Arabia; 3 Aramco Fuel Research Center, 232 Avenue Bonaparte, Rueil-Malmaison, 92852 France; 4 Present Address: Department of Machine Learning, Mohamed bin Zayed University of Artificial Intelligence (MBZUAI), Abu Dhabi, UAE

**Keywords:** Fossil fuels, Computational chemistry

## Abstract

High-performance fuel design is imperative to achieve cleaner burning and high-efficiency engine systems. We introduce a data-driven artificial intelligence (AI) framework to design liquid fuels exhibiting tailor-made properties for combustion engine applications to improve efficiency and lower carbon emissions. The fuel design approach is a constrained optimization task integrating two parts: (i) a deep learning (DL) model to predict the properties of pure components and mixtures and (ii) search algorithms to efficiently navigate in the chemical space. Our approach presents the mixture-hidden vector as a linear combination of each single component’s vectors in each blend and incorporates it into the network architecture (the mixing operator (MO)). We demonstrate that the DL model exhibits similar accuracy as competing computational techniques in predicting the properties for pure components, while the search tool can generate multiple candidate fuel mixtures. The integrated framework was evaluated to showcase the design of high-octane and low-sooting tendency fuel that is subject to gasoline specification constraints. This AI fuel design methodology enables rapidly developing fuel formulations to optimize engine efficiency and lower emissions.

## Abstract (web summary)

Practical liquid fuels involve hundreds of chemical species, making the prediction of mixture properties a key bottleneck for fuel design. Here, the authors develop an artificial intelligence framework to predict how interactions between molecules correlate with specific fuel properties and propose an optimized fuel mix.

## Introduction

The transport sector contributes to approximately a 25% of total global *CO*_2_ emissions. Note that >95% of transport energy originates from liquid hydrocarbon fuels^1^, primarily used to power combustion engines. There is a pressing requirement to lower transport-sector greenhouse gas and criteria pollutant emissions by developing more efficient powertrain technology and low carbon fuel formulations.

Engines’ environmental performance can be improved significantly by optimizing the fuel ignition quality and its sooting propensity. Engine knock, governed by fuel autoignition resistance, limits a spark-ignited engine’s ability to operate at its highest efficiency point. The research octane number (RON) and motor octane number (MON) are experimentally measured in cooperative fuel research engines at operating conditions according to ASTM standards^2,3^ and commonly used to assess fuel’s knocking behavior. Sooting propensity of a fuel is related to an engine’s particulate matter emissions. A high sooting fuel typically impacts engine efficiency through higher particulate filter backpressure^4^ and more frequent filter regenerations^5^ to achieve emission regulations. Various metrics have been proposed to characterize the chemical propensity of the fuel to form soot, including smoke point, threshold sooting index^6^, oxygen extended sooting index^7^, fuel equivalent sooting index^8^, etc. An alternative approach, the Yield Sooting Index (YSI)^9^, offers an advantage of more precise measurements for aromatics and is based on measurement of a maximum soot volume fraction. The formulation of fuels characterized by high knock resistance and low-sooting propensity could aid the transition to cleaner engines and fuels.

The traditional approach to fuel design is empirical and tedious, comprising (i) determining a potential blendstock, (ii) characterizing combustion-related properties of a candidate using experiments and simulations, and (iii) extensive research on understanding the effect of candidate's molecular structure on properties of the base fuel^10^. The challenges associated with this empirical approach reinforce the requirement for data-driven discovery of materials in multiple application areas, including clean energy, aerospace industry, and drug discovery^11^. Inverse fuel design is intrinsically distinct from the conventional approach. Rather than exhaustive parameter characterization from structures, the properties are selected beforehand, and new fuel candidates are obtained that match the requirements. In the inverse mode, the main driver for innovation is reverting mapping from structural information to properties.

The inverse fuel design problem is typically described as a constrained optimization task in which a mixture is formulated from a set of pure components in a chemical space to match the target properties. The corresponding workflow comprises two main parts: (1) accurate and rapid evaluation of chemical properties and (2) a robust and scalable search method to navigate in the chemical space and identify potential candidate mixtures. This two-step design process’s integrity can be ensured provided the tool offers a continuous and differentiable chemical space representation for various species; thus, it would allow direct optimization of properties using gradient-based methods. Here, machine learning (ML) algorithms, such as deep learning (DL)-based models, have a substantial advantage over other methods for inverse fuel design^12^.

DL has been successfully applied to cheminformatics and material science for tasks such as computing molecular properties, accurately predicting their interaction, and de novo generation of new molecules^13,14^. In the context of inverse design, generative models have been reported as promising tool for de novo molecule design using simplified molecular-input line-entry system (SMILES) representation and recurrent neural networks (RNN)^12^, a deep neural network architecture allowing modeling in the time domain. Several studies have been reported using ML to screen multiple combustion-related properties simultaneously on a molecular level^15,16^. The domain of applicability of these models covers a wide variety of hydrocarbon fuels, but they cannot be extended beyond pure molecules to encompass complex fuel mixtures. Screening mixtures instead of pure species is necessary to enable the discovery of novel combinations that improve fuel performance.

Because practical liquid fuels involve hundreds of species, the prediction of mixtures properties remains one of the key bottlenecks for the inverse fuel design. Algebraic mixing rules were proposed for iso-octane, n-heptane and toluene mixtures^17^, however, such approach is inapplicable to estimate properties of complex blends, e.g., containing oxygenates^18–25^. Alternatively, previously developed techniques for mixture screening mostly feature feed-forward networks with configurations unsuitable for the inverse design mode^26–29^. Note that details on the analysis of recent advancements in DL relevant to inverse fuel design are provided in the Supplementary Note 1.

To ensure eligibility of predictive model’s configuration for screening on a mixture level, it’s essential capability is an input representation applicable to pure components and mixtures. Moreover, mixing rules must be inherently implemented in the algorithm’s learning process to predict how interactions between molecules correlate with the specific property.

## Data-driven fuel design framework

**Fig. 1:**
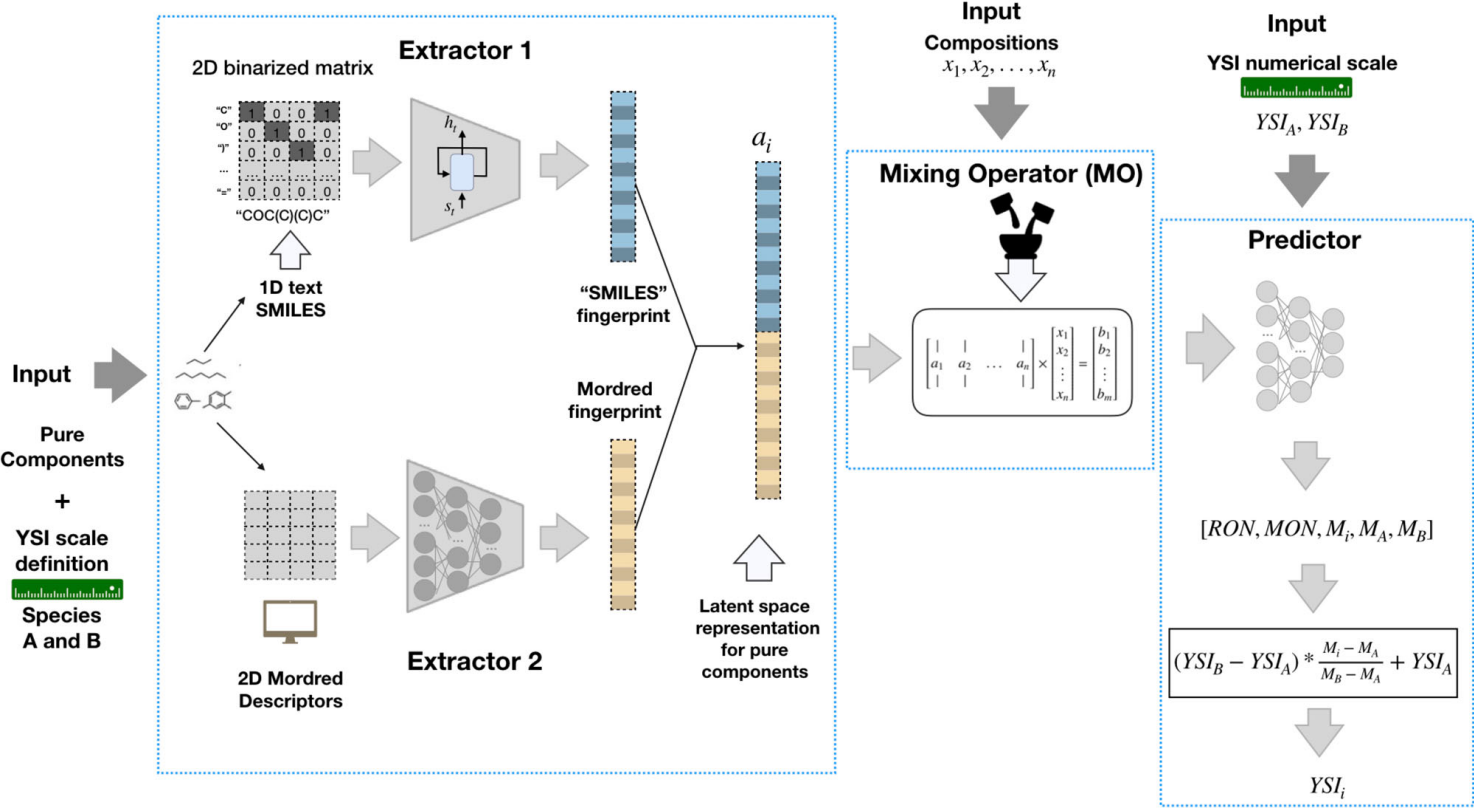
The network architecture of the joint-properties predictive model. The end-to-end DL model consists of four smaller parts: an encoder with recurrent layers (Extractor 1), an encoder with fully connected layers (Extractor 2), Mixing Operator (MO) and Predictor network. Detailed description is provided in Section ‘Predictive DL model’.

This work introduces a simple but elegant data-driven framework to inversely design fuels satisfying desired target properties. In particular, the AI fuel design tool was built on top of an end-to-end DL model based on recurrent and fully connected (FC) layers to predict three combustion properties of pure components and blends, namely, RON, MON, and YSI. Figure 1 shows a schematic of the entire network architecture. The curated database, on which the model was trained, contains single species from 19 molecular classes with oxygenates accounting for >20% of the pure species dataset, surrogate fuels mostly containing 2-10 pure components and complex mixtures, including gasoline.

We propose a linear mixing operator (MO) implanted into the training loop, the algorithmic advancement that enables direct connectivity between molecular and mixture representations, thus, fuel screening on a mixture level. In particular, the MO linearly combines latent vectors of pure components and their respective compositions to identify latent representation for mixtures.

The intuition behind MO is similar to the concept of embeddings in the latent space that are commonly used in Natural Language Processing. One such example is word2vec, which is an efficient technique for learning distributed vector representations of words that capture accurate syntactic and semantic word relationships^30^. Similarly, in our case a single word represents single species and mixtures correspond to phrases, which are a weighted combination of words in the hidden space. In addition to MO, we propose two robust and scalable search algorithms to navigate a well-defined chemical space and design fuels as mixtures satisfying constraints and target properties. The schematic diagram for the backward fuel design workflow is illustrated in Supplementary Fig. S1.

The evaluation demonstrated that the joint-properties predictive model acheives sufficiently high prediction accuracies while allowing for extracting latent representations for pure species and blends. We provide a complete evaluation of this fuel design framework across two tasks: the feed-forward predictive model and inverse design of the tool. First, we demonstrate model’s performance on the test set and compare it to multiple baselines. Then, formulated mixtures are analyzed to assess the proposed search approaches’ capability.

## Results and discussion

### Performance on the test set

**Fig. 2:**
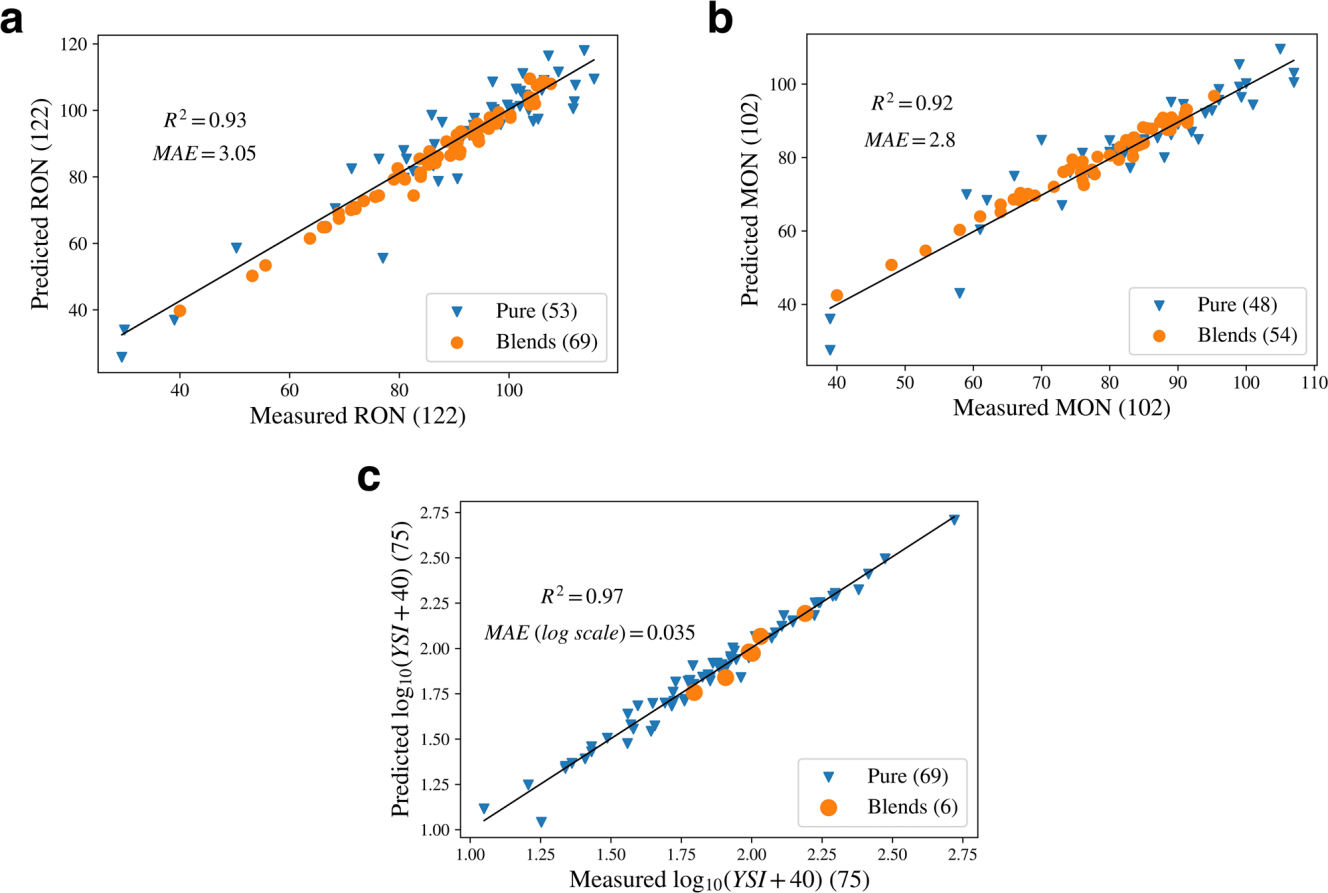
Performance of the joint-properties model on the test set. Parity plots show predictions on **a** RON, **b** MON and **c** YSI in log scale. Results shown for single components (blue triangles) and mixtures (orange circles).

We report proposed model’s performance in terms of coefficient of determination *R*^2^ and mean absolute error (MAE). Figure 2 shows the parity plots for the model’s independent test set, where model demonstrates acceptable performance by reaching *R*^2^ > 0.92 across all three target properties. Additionally, Table S1 in Supplementary Material reports *R*^2^ and MAE for each of the two inputs, i.e., single species and blends.

Looking at ON mapping, the model achieves higher prediction accuracy on mixtures, more represented in this database regarding single hydrocarbons. For the sooting index, the model shows good generalization capability in the target estimation of pure components on different numerical scales. Moreover, the model sustains its performance for YSI mixture predictions, although it is being the least represented data type in the database.

### Comparison with competing techniques

We compared the predictive model’s performance with (1) three data-driven models developed for predicting RON, MON, and YSI of pure components and (2) the linear-mixing rule for predicting ON of mixtures. The first baseline is the end-to-end learning model based on a graph neural network (GNN)^15^ developed for simultaneously predicting the derived cetane number (DCN), RON, and MON of single species. For a fairer analysis, the performance comparison was assessed on fifteen individual components from our test set that were excluded from the baseline model’s training set. Table 1 reports the resulting head-to-head analysis where the proposed model outperforms the baseline model with significant deviation on MAE on RON predictions. In addition, Table 1 compares the models’ performance on the respective full test sets. The proposed model shows a lower *R*^2^ coefficient for RON observations than the baseline model, and both models show similar *R*^2^ on MON data. However, MAE on RON predictions is similar for both models, whereas error on the proposed model’s MON predictions is slightly less than the GNN model. To summarize, both models demonstrate reasonably satisfactory performance, and the head-to-head analysis confirms the current model’s flexibility to predict the ON of a single species accurately.

**Table 1:** Comparison of RON/MON predictions with GNN model by Schweidtmann et al.^15^ on individual components from the test set of the proposed model, which were not included in the training set of GNN model, and on the entire test sets.

Compound name	True value		DL		GNN model^15^	
	RON	MON	RON	MON	RON	MON
3,4,4-Trimethyl-2-pentene	103	86.1	104.6	87.7	102.4	85.3
t-Butylbenzene	115.5	107.4	109.5	100.5	109.5	97.1
1-Methyl-2-N-Propylcyclohexane (trans)	29.4	38.8	25.7	27.5	39.1	40.1
2-Methylpentene-1	94.2	81.5	94.1	80.6	97.41	80
2-Methylpentene-2	97.8	83	99.3	84.3	96.7	82.9
1-Methyl-3-Propylbenzene	111.9	100.5	102.6	94.4	107.6	96.7
1,1,3-Trimethylcyclohexane	81.3	82.6	85.4	82.8	84.3	83.5
Methylfuran	103	86	102.6	87.6	107.2	96.8
Pentanol	103	90.8	103.2	94.5	102	92
4-Methylpentan-2-ol	102	95	101.3	92.8	99	91
Prenol	93.5	74.2	95.5	75.9	97	82
4-Methylene-1-(1-Methylethyl)bicyclo[3.1.0]hexane	80.9	59	79.7	70	86	71
7-Methyl-3-Methylene-1,6-Octadiene	82.5		81.7		90	
Dimethoxymethylbenzene	100.5		100.7		110	
(4R)-1-Methyl-4-(1- methylethenyl)- cyclohexane	87.1		78.7		89	
Mean absolute error (MAE)			2.68	4.04	4.23	4.54
*R*^2^ full test set			0.89	0.89	0.94	0.89
MAE full test set			4.7	3.8	4.5	4.4

To demonstrate accuracy in the ON predictions of blends, we report a performance comparison using the linear-by-mole mixing rule in Table 2. MAEs were calculated for the ON predictions of 69 mixtures of varying sizes in the independent test set. The algebraic mixing rule is a naive model regarding data-driven models. Nevertheless, this analysis provides a perspective on the proposed model’s MO’s generalization capability. To summarize, the model exhibits superior performance for blends across varied sizes when compared to naive baseline. Moreover, this model’s RON MAE decreases with the mixture size, whereas this qualitative trend is not evident with the naive baseline.

**Table 2:** Comparison on predictions of mixtures with linear mixing rule.

Mixture size	Count	DL MAE		Linear-by-mole mixing rule MAE	
		RON	MON	RON	MON
2	8	2.3	1.9	5.3	5.6
3	28	1.7	1.9	9.7	7
4	12	2.1	1.5	8	7.2
5	2	2.2		9.8	
6–10	3	1.1		2.8	
>10	16	1.3	2.1	6.9	4.8

Finally, Table 3 compares the proposed model’s YSI predictions to two baseline models. The artificial neural network (ANN) model^31^ was trained to fit the YSI of pure hydrocarbons measured on different numerical scales. The proposed model’s resulting median absolute error, *MedAE*, on the test set (69 species) is similar to the respective baseline model’s *MedAE* test sets (56 species). The second baseline, the quantitative structure-activity relationship (QSAR) model^32^, was trained on low soot scale data. The *MedAE* evaluated on 59 components is similar to the proposed model’s resulting *MedAE* on 43 test set components from the low soot scale. Table 3 reports *MedAE* on mixtures, slightly higher than *MedAE* for single components, explained by the scarcity of YSI measurements for blends in the training set. To summarize, the proposed model reaches decent performance on YSI predictions on multiple numerical scales.

**Table 3:** Comparison on YSI predictions classified by scale.

Scale	DL MedAE		ANN MedAE^31^	QSAR MedAE^32^
	pure (69)	mix (6)	pure (56)	pure (59)
All	4.58	5.9	4.34	
Low soot	2.9 (43)			3.08
Unified	4.84 (6)	5.65 (4)		
High soot	5.6 (20)	5.3 (1)		

### Analysis of obtained candidates

This section describes a post-screening analysis of potential fuel candidates obtained from the proposed data-driven fuel design framework. Building upon the shoulders of MO and features of generated mixture latent representation, we introduce the second part of the fuel design framework, a search tool described in Section ‘Exploring chemical space: Inverse fuel design’, to screen fuels on a mixture level. Since the latent space is a continuous and differentiable vector space, it allows direct gradient-based optimization of target properties. Based on this feature, two algorithms, full-scope search (see details in Section ‘Full-scope search’) and greedy search (see details in Section “Greedy” search’) have been proposed to formulate mixtures with desired target properties and that are subject to linear constraints. The search was performed for the target properties of RON = 95, MON = 85, and YSI = 60. Section ‘Constraints and targets’ describes the linear constraints, bounds, and details of the chemical space. From the results, 20 mixtures with 5-26 components were reported using a full-scope search, whereas the greedy search generated 66 mixtures with 3-6 components. These 86 fuel candidates exhibited three properties closest to the target RON, MON, and YSI values.

As a postprocessing step of obtained candidates, the Reid vapor pressure (RVP) of mixtures was estimated from their bubble pressure point at 37.8 ^o^C using the universal quasichemical functional group activity coefficient (UNIFAC) model with the Huron-Vidal mixing rule and Peng-Robinson equation of state. They were implemented as in the Phasepy library^33^, individual Antoine coefficients were extracted from the Yaws handbook^34^. Five of 86 mixtures exhibited RVP in an acceptable range (50 k*Pa* ≤ *RVP* ≤ 100 *kPa*).

Table 4 lists the five mixtures with an estimated lower heating value (LHV, Joback method), viscosity at 15 ^o^C (Saldana data^35^ for pure components with the Grunberg-Nissan mixing rule), and density (PubChem data for pure components with harmonic by-mass mixing rule^36^). Pie charts with the detailed composition of the five mixtures are provided in the Supplementary Material (Fig. S2).

**Table 4:** Short list of mixtures exhibiting acceptable RVP.

Mixture	RVP (kPa)	LHV (*M* *Jkg*^−1^)	viscosity (mPas)	density (*gcm*^−3^) at 15^*o*^*C*
17-1	78.7	38.2	1.11	1.04
22-13	50.3	40.3	1.36	0.751
18-17	67.4	39.7	0.77	0.791
26-20	52.5	37.8	1.20	0.785
6-1	61.0	42.4	0.65	0.732

Blend 17–1 shows a high density, preventing its use as drop-in gasoline. Mixture 22-13’s viscosity lies at the gasoline range’s higher boundary, indicating a higher Sauter droplet diameter than mixture 18-17.

For all mixtures, the Jaccard-Tanimoto similarity score^37^ for component pairs (based on RDKit fingerprints) demonstrates a log-normal distribution (provided in Supplementary Data 1), 12–13% of these component pairs exhibiting significant similarity (score > 0.5)^38^. In addition to multibranched paraffin (representing 16–36% liquid volume), all candidate mixtures contain C_1_-C_4_ alcohols (5–30%), C_5_-C_8_ cycloalkanes (3–35%), and C_4_-C_8_ alkenes (17–22%) conferring a high-octane sensitivity to the mixture. Note that the significant olefin content in the blends calls for autoxidation deposit and ozone formation potential assessment. In all blends, except for mixture 6-1, cycloalkenes (*cyclohexene*, *ethylidene*, and *cyclopentane*) and phenol ethers (*anisole* and *phenetole*) are present. Both families involve high-octane sensitivity compounds, put forward as potential octane boosters^39–41^. For certain mixtures (18-17, 26-20), primarily C_5_-C_6_ alkyl esters are present because lighter esters (such as *methyl acetate*) exhibit insufficient octane sensitivity. These esters should be compatible with fluorocarbon elastomers if used at low liquid fractions (<5%)^42^.

To summarize, the obtained mixtures illustrate that the proposed method can spot compounds previously identified in the fuel science community^43^ and suggest out-of-the-box gasoline components, screened at the mixture level. Among the new candidate components, *tetramethoxymethane*, primarily produced for pharmaceutical applications, can be discarded because its environmental properties stand beyond recommended limits^44,45^ (Supplementary Table S2). However, *isopentyl acetate*, a flavor enhancer previously not featured in transport applications, appears as a promising low-level component in gasoline blendstocks.

**Fig. 3:**
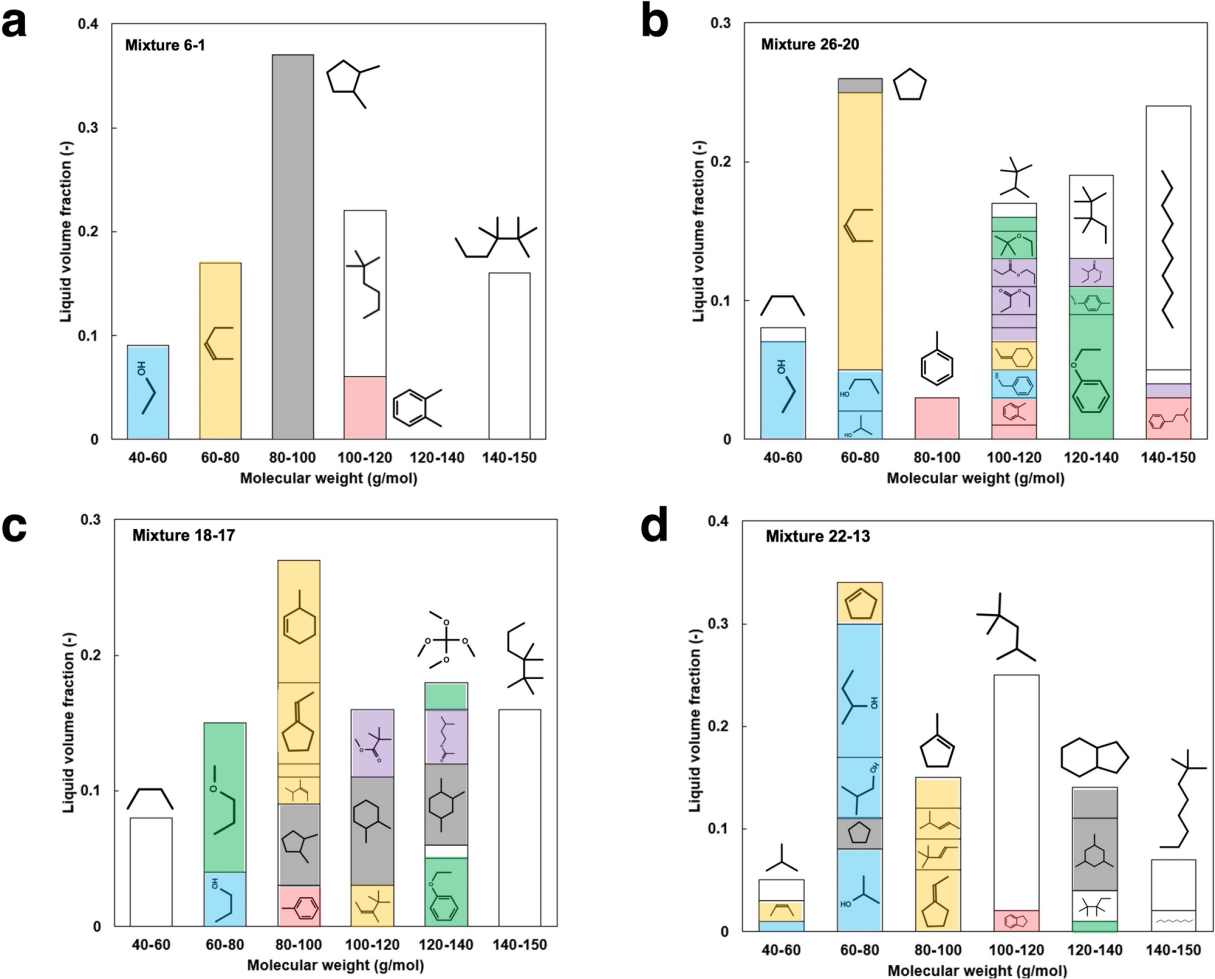
Molecular weight distributions of 4 mixture candidates with predicted RON/MON/YSI and mean squared error (mse) from the target properties. **a** mixture 6-1 with predicted properties 95/85/60 (mse = 0.00), **b** mixture 26-20: 94.7/85.4/59.8 (mse = 0.09), **c** mixture 18-17: 95.4/85.1/59.6 (mse = 0.09), **d** mixture 22-13: 94.9/85.3/59.6 (mse = 0.07). Components were classified by families of species: alcohols (blue), aromatics (red), esters (purple), olefins (orange), paraffins (white), cycloalkanes (grey) and ethers (green).

Component molecular weight distributions reveal a near mono-mode at 100 g/mol, except for mixtures 26-20 and 22-13, showing a marked multimodal molecular weight distributions (Fig. 3). This idiosyncratic distribution should impact gas-phase composition distribution in the combustion chamber and reactivity at the gasoline spray periphery^46,47^. Based on previous studies^48^, light alcohols should first evaporate, enhancing knock resistance through high heat of vaporization. Alkenes and cycloalkanes would evaporate in turn, followed by C_5_-C_6_ esters and finally large alkanes and phenol ethers. Blend 26-20 has a greater fraction of heavy components (140–150 g/mol range) in comparison to 22-13, implying likely higher levels of unburnt hydrocarbon emissions at the exhaust.

The analysis concluded that mixture 22-13 is the most promising candidate for a 95/85/60 gasoline blendstock. Individual component availabilities and mixture cold-weather performance should be studied to characterize the drop-in character on such a blendstock fully.

## Conclusion

This work introduced a conceptually simple and fully data-driven framework to design fuel mixtures matching desired combustion-related properties, enabling high-octane mixtures and low engine-out soot emissions. The proposed workflow comprises the joint-property predictive model and search approach. In the first step, the feed-forward network with RNN and FC layers was trained on a comprehensive database, including RON, MON, and YSI measurements from the literature of single species from 19 molecular classes, surrogate fuels, and complex mixtures. The innovative part of the network is an adaptation of MO, a mechanism generating mixture-hidden representation by performing linear combinations on hidden vectors of pure components. MO enables using a mixture’s detailed composition and species’ molecular information as a direct input to the model without preprocessing. The extensive assessment of the DL model with competing baselines for pure components across diverse modalities demonstrates that the proposed model consistently performs reasonably well on the unseen test set. Moreover, the proposed model achieves 93–96% accuracy for *R*^2^ on mixtures for three properties.

The features of MO used jointly with the direct computational graph’s structure in the neural network allowed formulating the fuel design problem to solve it using a standard optimization technique. Here, full-scope and greedy search methods were proposed to identify suitable mixtures in the chemical space. The former generates mixtures with 5-26 components, whereas the latter formulates blends with fewer components. Using the proposed workflow, 86 gasoline candidates were determined with target RON = 95, MON =  85, and YSI = 60 properties. After additional screening with RVP, density, viscosity, and LHV, one mixture containing 22 components was preserved as the most promising candidate. In future work, we plan to extend the current database by curating other relevant properties, such as RVP, viscosity, density, and LHV, essential criteria in fuel screening. Ultimately, future comparison of short-listed mixtures experimental properties with predictions will allow us to further improve tool accuracy and valorize the current framework. Moreover, future workflow versions should preclude formulations based on component availability. We expect our simple and practical framework will serve as a solid baseline and help ease future research designing liquid energy carriers.

## Methods

### Data curation

The database of experimentally obtained measurements for the three combustion-related properties (RON, MON, and YSI) for single hydrocarbons and mixtures was curated from many literature sources. Table 5 summarizes the elements in the collected database, where the entire dataset is classified into three subpopulations: pure components, ≤ 10-component blends (mostly surrogates), and complex fuels with more than 10 components.

**Table 5:** Summary of classes in the curated ON and YSI database for predictive model development.

	Class	RON	MON	YSI
Pure components	n-alkanes	7	7	8
	iso-alkanes	44	42	22
	alkenes	86	83	36
	cycloalkanes	75	64	16
	cycloalkenes	22	22	14
	aromatics	45	42	131
	alkynes	8	4	4
	terpenes	3	2	0
	NON-Oxygenates	290	266	231
	alcohols	23	19	46
	ethers	6	6	14
	esters	18	17	64
	other cyclic ethers	2	2	4
	ketones	10	9	28
	cyclic ketones	2	2	2
	aldehydes	0	0	17
	furans	3	3	6
	hydrofurans	3	3	6
	acetals	1	1	0
	lactones	1	1	0
	other multi-oxygen compounds	6	4	33
	Oxygenates	75	67	220
	total pure components	365	333	451
Mixtures	*with* ≤10 *components*	372	293	35
	*with* >10 *components*	76	64	5
	total mixtures	448	357	40
Total		813	690	491

We extended the RON/MON pure components database published by Schweidtmann et al.^15^ and the Yale University YSI database^49,50^ by adding measurements for oxygenated compounds^51,52^. The curated data include data for single species from 19 molecular classes with 365 RON, 333 MON, and 451 YSI observations. More interestingly, oxygenated compounds account for approximately 20% and 50% of single component ON and YSI databases, respectively.

For mixtures with 2-10 components, ON data were collected for 372 blends, including 22 hydrocarbon representatives from five molecular classes (n-alkanes, isoalkanes, alkenes, cycloalkanes, and aromatics) blended with five oxygenated hydrocarbons, four alcohols (methanol, ethanol, 2-propanol, and prenol), and MTBE^19,27,53–57^. Furthermore, detailed hydrocarbon analysis and ON measurements, characterizing an ignition quality of 76 real fuels, were extracted from the literature. These complex mixtures include 30 fuels for advanced combustion engines (FACEs) mixed with ethanol^58–61^, Haltermann and Coryton gasoline fuels^62^, 3 FACE F + *terpineol*^23^, 36 reformulated blendstocks for oxygenated blending blended with prenol/other *C*_5_ alcohols^63^, and five test gasoline fuels^64^.

The literature scarcely reported YSI measurements for mixtures for the third property database. Overall, only 40 measurements of the sooting index for mixtures and their detailed compositions were found. These values were identified for diesel, gasoline, and jet fuel surrogates^65–67^ and co-optima test gasoline and its surrogates^68^. YSI quantification is based on measurement of a maximum soot volume fraction (*M*_*i*_) directly proportional to the sooting propensity. *M*_*i*_ is measured on the centerline of a coflow methane/air nonpremixed flame doped with 400 ppm test fuel and converted to an apparatus-independent YSI using the following equation^69^:

1 $$YS{I}_{i}=(Y\,S{I}_{B}-YS{I}_{A})* \frac{{M}_{i}-{M}_{A}}{{M}_{B}-{M}_{A}}+YS{I}_{A}$$

where A and B are the two reference compounds. In analogy to octane rating, the numerical scale, which is used to translate the measured quantity *M*_*i*_ to YSI, is defined by lower and upper endpoint species and the values assigned to them, *YSI*_*A*_ and *YSI*_*B*_. Multiple numerical scales were reported in the literature to accurately assess the YSI of hydrocarbons whose sooting propensity is too different to capture in a single experimental setup. Four ratings were identified in measurements of the curated YSI database, and the summary is shown in Supplementary Table S3. Furthermore, different experimental techniques were used to measure *M*_*i*_ quantity, including color-ratio pyrometry, light extinction measurement, and laser-induced incandescence. Supplementary Fig. S3 depicts the data distribution histograms for ON and YSI databases.

### Train and test split

In Table 5, the curated database contains values of three properties for pure components and mixtures; however, only 141 data points have all three measurements available, and the remaining 1018 observations have at least one missing property. The customized hierarchical stratified sampling was used to split the dataset to ensure that observations from all relevant subpopulations were included in the training/validation and testing sets. The entire population was divided into six nonoverlapping subsets based on the availability of specific properties, e.g., Subset 1 (Sub 1) contains observations with all three properties (RON, MON, and YSI). Two nonoverlapping strata were defined within each subset: single species and mixture observations. Next, each subset was randomly split into 85% train/validation and 15% test set using stratified sampling in the scikit-learn library^70^ to ensure 15% of each stratum (pure species and mixtures) was randomly sampled into the test set. Each subset’s final train and test sets were merged, and Table S4 in Supplementary Material reports the resultant datasets. Training and test datasets for pure components and mixtures are provided in Supplementary Data 2 and Supplementary Data 3.

### Predictive DL model

#### Molecular representation

As a molecular input to the predictive model, we used a one-dimensional text representation, SMILES strings, with molecular descriptors calculated using the Mordred platform^71^. SMILES nomenclature is based on small and natural grammar, providing rigorous structure notation derived from molecular graph theory principles^72^. SMILES strings are widely used to represent molecules for chemical information processing tasks, such as property prediction and inverse molecular design. Aromatic SMILES were identified for 649 pure species using the Chemical Identifier Resolver tool developed by the National Cancer Institute^73^. Mordred is an open-source molecular-descriptor-calculation software generating more than 1800 2D and 3D descriptors. Generated SMILES strings were converted to a binary matrix using one-hot encoding. As data preprocessing step, Mordred descriptors were normalized using a min-max scaler in an open-source ML library scikit-learn^70^. More specifically, descriptors of the pure components in the unseen test set were normalized based on the scaling factors fitted on the species in the training set’s descriptors.

#### Network architecture

The end-to-end DL model incorporates three smaller networks (Extractor 1, Extractor 2, and Predictor) and an MO (see Fig. 1). The proposed model structure is conceptually simple. The molecular fingerprint is encoded via Extractor 1 and Extractor 2, the MO generates mixture fingerprints based on linear operation, and the predictor maps fingerprints to the target properties.

#### Extractor 1 and Extractor 2

To take advantage of the sequential nature of the text representation and allow dependence modeling through sequence between each character in a SMILES string, we used one of the RNN’s architecture, namely, the long-short-term memory (LSTM) cell^74^. Compared to conventional feed-forward neural network architecture, RNNs include a specific unit in architecture called memory blocks in recurrent hidden layers.

The proposed Extractor 1 architecture includes three stacked LSTM layers with the descending dimensionality of output features. Thus, LSTM Encoder extracts the most informative features from SMILES string to a vector, referred here as ’SMILES fingerprint’. Extractor 2 maps Mordred descriptors to a Mordred fingerprint. It includes three sequential FC layers with a rectified linear activation function used as output units and the last FC layer with linear hidden units. In the next layer, two fingerprints are concatenated along the second dimension into a vector referred to as a latent or hidden space representation for pure components, denoted as *a*_*i*_. The parameters of Extractors 1 and 2 are trained to transform the original data, molecular information, into another representation, a vector with the most semantic features for predicting joint properties.

#### Mixing operator (MO)

Another essential design consideration is defining latent space representation for mixtures, which can be directly used to predict target properties of the given blend. Here, hidden space representation of a mixture is defined as a linear combination of single component vectors based on their respective compositions. This definition can be expressed as a matrix-vector multiplication performed in a MO:

2 $$\left[\begin{array}{cccc}| &| &&| \\ {a}_{1}&{a}_{2}&\ldots &{a}_{n}\\ | &| &&| \end{array}\right]\times \left[\begin{array}{c}{x}_{1}\\ {x}_{2}\\ \vdots \\ {x}_{n}\end{array}\right]=\left[\begin{array}{c}{b}_{1}\\ {b}_{2}\\ \vdots \\ {b}_{m}\end{array}\right],$$

where *m* is the dimension of the latent space vector, $A\in {{\mathbb{R}}}^{m\times n}$ is a matrix containing latent vectors *a*_*i*_’s of *n* single species, ${{{{{{{\boldsymbol{x}}}}}}}}\in {{\mathbb{R}}}^{n}$ with $\mathop{\sum }\nolimits_{i = 1}^{n}{x}_{i}=1$ and *x*_*i*_ ≥ 0 for all *i* ∈ {1, 2, …, *n*} is a vector of respective compositions of *n* pure components, and ${{{{{{{\boldsymbol{b}}}}}}}}\in {{\mathbb{R}}}^{m}$ is the resultant latent representation of the mixture.

#### Predictor

The latent vectors, generated from Extractors 1 and 2 for single species and the MO for mixtures, are further processed using the predictor network that maps fingerprints to the three combustion-related properties. The predictor network comprises three FC layers with rectified linear activation functions and a final linear layer.

Several numerical scales in the curated YSI database (Supplementary Table S3) can be an additional bottleneck in modeling the joint-property predictive model. Therefore, to extend the model’s capability to evaluate YSI on any given scale, we predict *M*_*i*_, *M*_*A*_ and *M*_*B*_ from the molecular structural information of the test fuel (i) and two reference compounds (A and B). The last step is postprocessing predictions and calculating the test fuel’s YSI value using Eq. (1). Therefore, the model’s input includes SMILES, Mordred descriptors of pure components, compositions for mixtures, and the scale on which YSI is estimated, namely, SMILES and Mordred descriptors of lower and upper endpoint species (A, B) and their assigned YSI values.

Since the scale-space of the three output variables is significantly different (Supplementary Fig. S2) and the error function (MSE loss) is scale-sensitive, the weighted loss function is used to train the model.

The proposed model’s architecture exhibits numerous hyperparameters to be tuned, including batch size (*B*), learning rate (*lr*), predictor architecture, and Extractors 1 and 2. The final output sizes of the latter two determine the optimal dimension of the latent space vector (*m*) for pure components and mixtures. The hyperparameter tuning was performed using an adaptive experimentation platform using the Bayesian optimization algorithm (https://ax.dev/). The optimal hyperparameter settings were based on the validation set, comprising 15% of the training set. After the tuning, the optimal hidden vector dimension was 24 (i.e., *m* = 24), reported in Supplementary Table S5 with the other parameters.

### Exploring chemical space: Inverse fuel design

Our primary objectives with the search tool are to design mixtures that

- match target RON, MON and YSI,
- are subject to known physical constraints, e.g. gasoline specifications, and
- are of widely varying size, i.e., different number of blendstocks in a mixture.

To match these goals, we propose a full-scope search, a search procedure performed on the entire chemical space generated from the available database. During iterative testing, it was observed that the full-scope search tends to find optimal solutions, i.e., mixtures, containing anywhere between 5-26 single components. This may be caused by the high dimensionality of the search problem since in this study the chemical space was mapped by 514 pure components. Unfortunately, sparsity in the output solution vector *x* cannot be directly enforced as sparsity-enforcing penalties such as *ℓ*_*∞*_ or *ℓ*_1_ norms^75^ cannot be formulated as a vectorized linear function as required in Eq. (3). To offer the ability to obtain fuels of smaller sizes, we propose the second search approach, the greedy search. This approach exploits the solutions found by the full-scope search and reduces them in size to find mixtures with potentially fewer components, e.g., three to six pure species.

#### Full-scope search

Since DL is a form of a feature learning based on the nonlinear mappings and the resulting problem is highly non-convex, we first provide a set of *k* candidates which are starting points for the search. These are chosen as the closest points from the curated database (Table 5) to the vector in the latent space corresponding to the target properties, where the distance is defined as the MSE. The pseudocode for candidate search is provided in Table 6.

**Table 6:** Algorithm 1: Candidates Search.

	**Inputs:** the number of candidates *k*, target ***y***
1:	**for** each observation ***o***_*i*_ from database; *i* = 1, , …do
2:	Calculate: ${{{{{{{{\rm{loss}}}}}}}}}_{i}={\left\Vert {{{{{{{\rm{model}}}}}}}}({{{{{{{{\boldsymbol{o}}}}}}}}}_{i})-{{{{{{{\boldsymbol{y}}}}}}}}\right\Vert }^{2}$
3:	**end for**
	**Output:** set of *k* candidates with smallest loss_*i*_

We subsequently describe the optimization workflow for the full scope search, further documented in Algorithm 2 in Table 7. The objective is to find a set of optimal composition vectors denoted by ***x***^⋆^, which can be written as the following optimization problem

3 $$\begin{array}{rcl}{{{{{{{{\boldsymbol{x}}}}}}}}}^{\star }&=&\arg \mathop{\min }\limits_{{{{{{{{\boldsymbol{x}}}}}}}}\in {{\mathbb{R}}}^{n}}{\left\Vert {{{{{{{\rm{Predictor}}}}}}}}(A{{{{{{{\boldsymbol{x}}}}}}}})-{{{{{{{\boldsymbol{y}}}}}}}}\right\Vert }^{2}\\ &&{{{{{{{\rm{s.t.}}}}}}}}\;{{{{{{{{\boldsymbol{c}}}}}}}}}^{l}\le c({{{{{{{\boldsymbol{x}}}}}}}})\le {{{{{{{{\boldsymbol{c}}}}}}}}}^{u};{{{{{{{{\boldsymbol{x}}}}}}}}}^{l}\le {{{{{{{\boldsymbol{x}}}}}}}}\le {{{{{{{{\boldsymbol{x}}}}}}}}}^{u},\end{array}$$

where *c*(***x***) is a vectorized linear function with its upper and lower bound, e.g., $\mathop{\sum }\nolimits_{i = 1}^{n}{x}_{i}=1$ is encoded here. ***x***^*u*^ and ***x***^*l*^ are the upper and lower bounds for the composition vector, respectively. Matrix *A* contains a latent representation of all pure components as columns, therefore, *A****x*** refers to the mixture’s representation (see (2)). Vector ***y*** contains target properties as entries. We solve this problem using optimize subpackage in the open-source scientific Python computing library—scipy^76^. We call the optimize function *k* times, each time with a different starting point, which were obtained using Algorithm 1 in Table 6. We output all the possible solutions with a loss smaller than the given threshold *ϵ*. The pseudocode is provided below.

**Table 7:** Algorithm 2: Full-scope search.

	**Inputs:** target ***y***, the number of starting points *k*, *Predictor*( ⋅ ), latent vectors matrix *A*, constraints *c*( ⋅ ), ***c***^*u*^, ***c***^*l*^, ***x***^*u*^, ***x***^*l*^, threshold *ϵ*
1:	Obtain starting points {*s*_1_, …, *s*_*k*_} using Alg. 1
2:	**for** each starting point ***s***_*i*_; *i* = 1, 2, …*k* **do**
3:	${{{{{{{{\boldsymbol{x}}}}}}}}}_{i}^{\star }=optimize({{{{{{{{\boldsymbol{s}}}}}}}}}_{i},c(\cdot ),{{{{{{{{\boldsymbol{c}}}}}}}}}^{u},{{{{{{{{\boldsymbol{c}}}}}}}}}^{l},{{{{{{{{\boldsymbol{x}}}}}}}}}^{l},{{{{{{{{\boldsymbol{x}}}}}}}}}^{l})$, see (3).
4:	**end for**
	**Output:**${{{{{{{\mathcal{X}}}}}}}}={\left\{{{{{{{{\boldsymbol{{x}}}}}}}_{i}^{\star }}};{\left\Vert {{{{{{{\rm{Predictor}}}}}}}}(A{{{{{{{{\boldsymbol{x}}}}}}}}}_{i}^{\star })-{{{{{{{\boldsymbol{y}}}}}}}}\right\Vert }^{2}\le \epsilon \right\}}_{i\in \{1,\ldots ,k\}}$

For more efficient optimization, we provide an optimizer with a gradient that can be efficiently calculated and extracted using the automatic differentiation module of PyTorch^77^, a Python open-source library used for implementing DL architecture. The automatic differentiation library provides a functional interface, tracking tensors and all performed operations in a directed acyclic graph, where inputs are leaves, and output tensors are the roots.

#### "Greedy" search

To generate mixtures of reduced size, e.g., 3–6 components, we adopt a “greedy” search based on the traversal depth-first search algorithm^78^, where the tree’s root is the initial mixture *M* found from the general solution with many number of components. The tree’s nodes are generated by removing components individually as shown on the left side of the diagram and rescaling the composition by satisfying the constraints. If the constraints are satisfied, the node is added to the graph. The constraints were matched using Dykstra’s method to compute a point in the intersection of convex sets^79^. Depth-first search recursively conducts an exhaustive search of all nodes by proceeding, if possible, else to backtrack to the neighbors of all upper levels until the solution is found. Visualization of this search approach is illustrated in Supplementary Fig. S4.

#### Constraints and targets

The chemical space was limited to the CHO space to evaluate the search tool. The following criteria were used to exclude species from the search:

- alkynes (sooting components), aldehydes (unstable components)
- components with molecular weight outside of range 45–150 g/mol,
- sooting components with more than one aromatic ring,
- sooting components with aromatic ring and and extra unsaturation (e.g. *styrene*, *indene*),
- sooting components with more than 3 unsaturations (excluding aromatic, e.g. *octatetraene*)

In this work, the considered chemical space includes only molecules that were present as a single component or part of blends in the curated database. However, in general, any new pure component outside of the database can be added to the chemical space to design mixtures by providing molecular information in terms of SMILES string and molecular descriptors generated by Mordred, i.e., the inputs to Extractor 1 and 2, without need to know its experimentally measured properties.

The default linear constraint corresponds to the requirement that the sum of the compositions in a given mixture, *x*, must be equal to one $\mathop{\sum }\nolimits_{i = 1}^{n}{x}_{i}=1$. Other requirements in implementing the search approach follow European gasoline specifications^80^ and are summarized in Table S6 in Supplementary Material. An additional constraint was specified to consider a maximum volume threshold (10%) for the transporting fuels containing 3, 4, 7 and 8 aliphatic rings (saturated and unsaturated).

To identify promising gasoline blends, *RON* = 95, *MON* = 85 and *YSI* = 60 target values were screened. The YSI was estimated on a ‘unified” scale, Table S3 in Supplementary Material provides details on YSI scales.

## Supplementary information

Supplementary Material

Description of Additional Supplementary Files.docx

Data Set 1

Data Set 2

Data Set 3

## Data Availability

The authors declare that the data supporting the findings of this study are available within supplementary information files.

## Code availability

Codes developed in this work can be available upon reasonable request to the corresponding author via e-mail.
