# Supplementary material for: Artificial intelligence-driven design of fuel mixtures
Source: Commun Chem. 2022 Sep 16;5:111. doi: 10.1038/s42004-022-00722-3 (PMC9814251; doi:10.1038/s42004-022-00722-3)
Supplement: Supplementary file 1 — Supplementary Material [file 42004_2022_722_MOESM1_ESM.pdf]

Artificial intelligence-driven design of fuel mixtures: Supplementary material

Supplementary Note 1

Over the last decade, DL outperformed AI technologies based on other ML algorithms, the advantage of DL is the flexibility of neural network architecture, which can be tailor-made for the specific task. It achieves great success by learning to represent input as a nested hierarchy of concepts, with more abstract representations derived from less abstract ones [1]. DNN parameters are trained to transform original data into another representation which is a vector with the most semantic features for the prediction of joint properties, thus in this respect, DL is a form of feature learning based on the non-linear mappings. This intermediate vector is referred to as a latent space or hidden space representation, which is continuous and differentiable [2]. For a task of designing fuel matching not one, but multiple combustion-related properties, it is crucial that the latent vector of each datum is consistent and sufficiently informative to predict all properties of interest. Merging databases of different properties helps to fill in gaps in hidden space, thus, widens the chemical space, however it may also arise issue with respect to missing data.

Prior works have been reported to apply ML methods to predict multiple combustion-related properties of single components. For example, Schweidtmann et al.[3] proposed Graph Neural Network (GNN) that maps molecular graph to the three ignition quality indicators, the Derived Cetane Number (DCN), RON and MON. A single neural network model was trained to predict multiple thermochemical properties and used to screen high energy components [4]. These models can be used to screen a wide variety of hydrocarbon fuels, but its applicability cannot be extended to screening on a mixture level.

The prediction of mixture properties remains also a challenge of great importance in the inverse fuel design since the composition of fuels can be complex. A simple linear-mixing-by-mole rule was proposed by Knop et al. [5] to estimate octane numbers based on values of pure species and results demonstrated high accuracy of RON and MON predictions for n-heptane/iso-octane/toluene mixtures. However, there is a growing evidence that octane enhancers, such as some oxygenates, do not follow a linear mixing rule and can have both synergistic or antagonistic effect on overall ignition quality depending on the composition and properties of the base fuel and the blendstock. These studied octane boosters include bioethanol [6–10], prenol [11], terpineol [12], furan mixtures, i.e. methylfuran and dimethylfuran)] [13, 14].

In addition, mixture properties are currently evaluated through mixing rules at the descriptor [15] or physical properties [16] level. Alternatively, a lot of research effort has been invested with the goal finding the most informative representation for mixtures, which can be directly used as an input without incorporation of mixing rules. For example, the input based on measurements

obtained via various analytical techniques was shown to be efficient in predicting ONs of complex mixtures, these techniques include flame spectroscopy emission [17], Near-Infrared (NIR) Spectroscopy [18], gas chromatography [19], nuclear magnetic resonance (NMR) spectroscopy [20, 21], ignition quality tester (IQT) [22]. Data from these methods were mapped into RON and MON using partial least-squares [17, 18], multilinear regression (MLR) [20], artificial neural networks[21, 23]. Recent joint Birmingham-Shenzhen work [24] featured a machine learning approach to predict 15 different properties, mixtures properties being obtained through functional group interaction. The mixture representations used in these models cannot be directly connected to the representations of pure components, thus, the configuration of these models is ineligible for inverse fuel design.

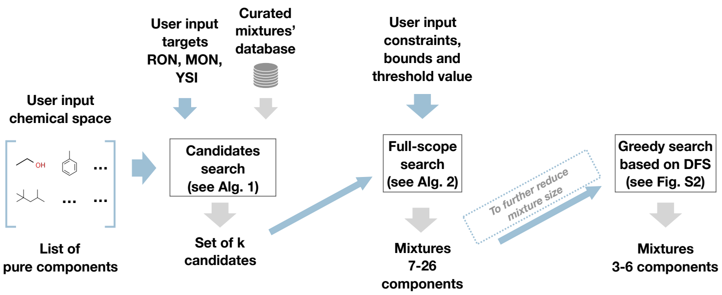

Fig. S1: Backward fuel design workflow.

Table S1: Performance of the proposed model on the test set.

Property		R^2	MAE
RON	pure	0.89	4.73
	mix	0.98	1.76
	all	0.93	3.05
MON	pure	0.89	3.85
	mix	0.96	1.85
	all	0.92	2.8
YSI	pure	0.99	4.58
	mix	0.95	5.9

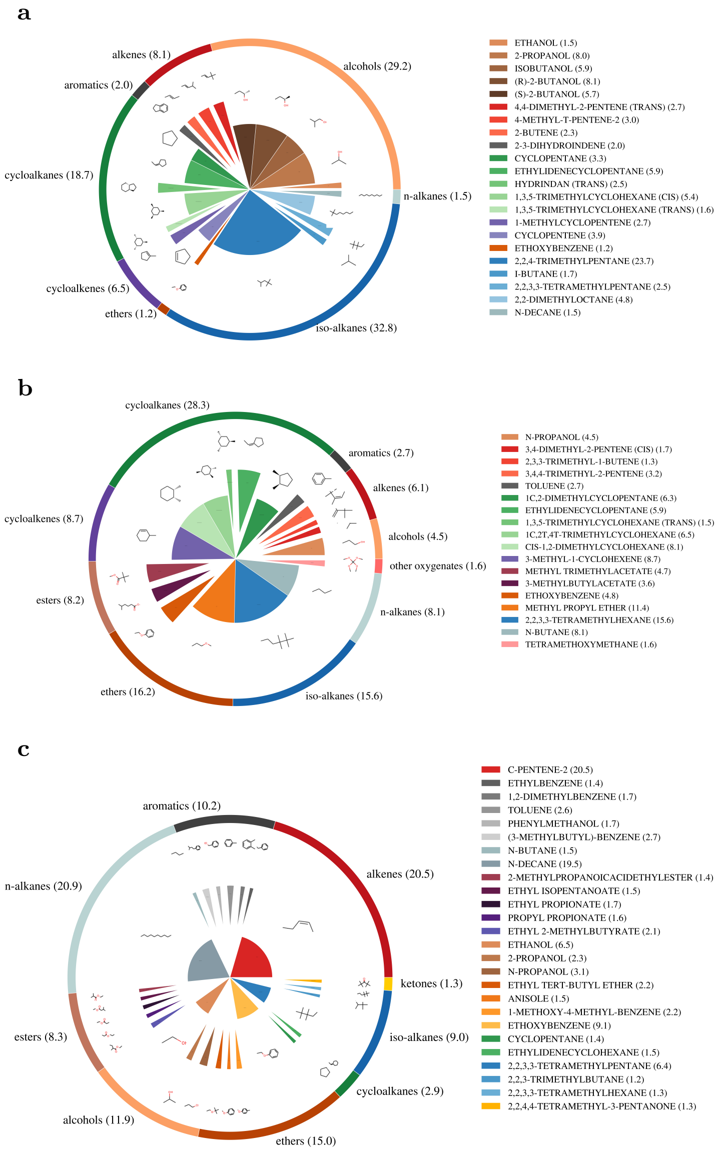

Fig. S2: Pie charts of final 4 candidates with predicted $RON/MON/YSI$ value and mean squared error from the target properties. **a** mixture 22-13 with properties 94.9/85.3/59.6 (mse = 0.07), **b** mixture 18-17: 95.4/85.1/59.6 (mse = 0.09), **c** mixture 26-20: 94.7/85.4/59.8 (mse = 0.09).

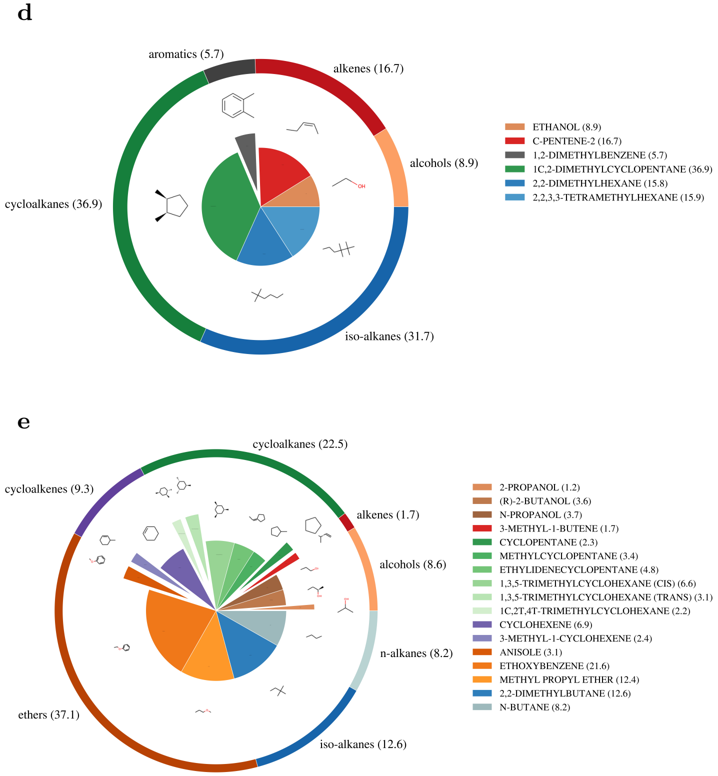

Fig. S2: Pie charts of final 4 candidates with predicted $RON/MON/YSI$ value and mean squared error from the target properties (cont.). **d** mixture 6-1: 95/85/60 (mse = 0.00), **e** mixture 17-1: 95/85/60 (mse = 0.00).

Table S2: Supplementary properties of new potential components from short-listed mixtures.

**Compound**	FKMC	EPIB7	WS (gL^{-1})	AHL (d)	MP (^{o}C)
Tetramethoxy methane	yes	-0.845	110	3.2	-5
Isopentyl acetate	yes (if $< 5\%$)	0.655	2.0	1.8	-78
Ethylidene cyclopentane	yes	0.203	0.1	0.12	-129

*Notation: FKMC = FKM compatibility, EPIB7 = EPI Biowin 7, WS = Water solubility, AHL = Atmospheric half-life, MP = Melting point.

Table S3: Numerical scales in YSI measurements.

Scale	Lower endpoint	Upper endpoint	YSI_A	YSI_B	Species	Refs
	n-heptane	tetralin	10	100	diesel surrogates	[25]
low soot	n-hexane	benzene	0	100	pure species, diesel and jet fuel surrogates	[26, 27] [28]
high soot	benzene	1,2-dihydro-napththalene	30	100	pure species	[29, 30]
	benzene	toluene	30	43.5	gasoline surrogates	[31]
unified	n-hexane	benzene	30	100	pure species	[28]
	n-heptane	toluene	36	170.9	TG and surrogates	[32]

Table S4: Train/test split with hierarchical stratified sampling.

Subset #	strata	train	test	total
Sub 1	pure	115	21	136
RON, MON and YSI	mix	4	1	5
Sub 2	pure	159	28	187
RON and MON	mix	294	53	347
Sub 3	pure	5	1	6
RON and YSI	mix	0	0	0
Sub 4	pure	23	4	27
RON	mix	81	15	96
Sub 5	pure	260	47	307
YSI	mix	30	5	35
Sub 6	pure	0	0	0
MON	mix	5	0	5

Table S5: Tuned hyperparameters.

Net	Parameter	Value
	Optimizer	ADAM
	Batch size	64
	Learning rate	0.001
	Latent vector dim (m)	24
Extractor 1	# LSTM layers	3
	hidden states dim	[256, 64, 16]
	nodes dim in FC layer	12
Extractor 2	# FC layers in	3
	nodes dim	[1048, 512, 12]
Predictor	# FC layers	
	nodes dim	[128, 24, 12]

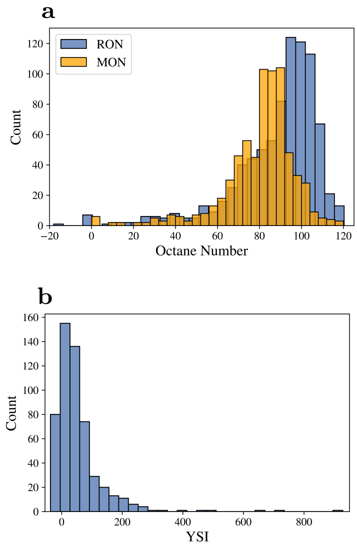

Fig. S3: Distribution of target values in database. Histograms shown for **a** RON and MON , and **b** YSI.

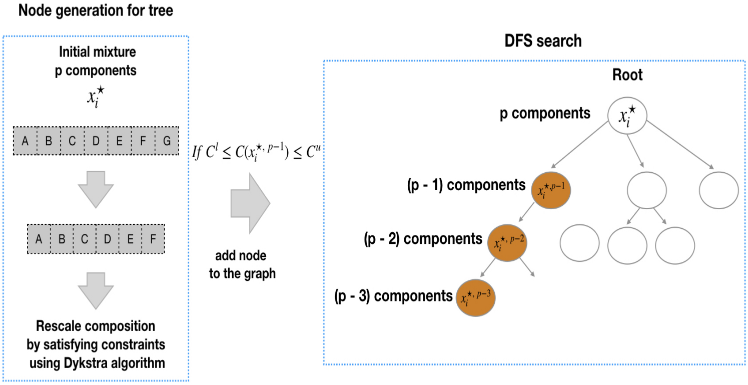

Fig. S4: Depth-first search algorithm.

Table S6: Gasoline specifications used as linear constraints.

Species category	Upper threshold (% v/v)
Aromatics	35
Benzene	1
Olefins	18
Diolefins	1
Ethers	22
Cyclic ethers, furans and hydrofurans	1
Ethanol	10
Methanol	3
Isopropanol	12
Butanol	15
Other oxygenates	15
All Oxygenates	30
C_3, C_4, C_7 and C_8 aliphatic rings	1

Supplementary References

[1] Bengio, Y., Goodfellow, I. & Courville, A. *Deep learning* Vol. 1 (MIT press Cambridge, MA, USA, 2017).

[2] Schwalbe-Koda, D. & Gómez-Bombarelli, R. *Generative Models for Automatic Chemical Design*, 445–467 (Springer International Publishing, Cham, 2020). URL https://doi.org/10.1007/978-3-030-40245-7_21.

[3] Schweidtmann, A. M. *et al.* Graph neural networks for prediction of fuel ignition quality. *Energy & fuels* **34** (9), 11395–11407 (2020) .

[4] Li, G. *et al.* Machine learning enabled high-throughput screening of hydrocarbon molecules for the design of next generation fuels. *Fuel* **265**, 116968 (2020) .

[5] Knop, V., Loos, M., Pera, C. & Jeuland, N. A linear-by-mole blending rule for octane numbers of n-heptane/iso-octane/toluene mixtures. *Fuel* **115**, 666–673 (2014). URL https://www.sciencedirect.com/science/article/pii/S0016236113006923. https://doi.org/https://doi.org/10.1016/j.fuel.2013.07.093 .

[6] Anderson, J. E. *et al.* Octane numbers of ethanol-gasoline blends: measurements and novel estimation method from molar composition. Tech. Rep., SAE Technical Paper (2012).

[7] Foong, T. M. *et al.* The octane numbers of ethanol blended with gasoline and its surrogates. *Fuel* **115**, 727–739 (2014) .

[8] Aronsson, H. S., Tuner, M. & Johansson, B. Using oxygenated gasoline surrogate compositions to map ron and mon. Tech. Rep., SAE Technical Paper (2014).

[9] Hirshfeld, D. S., Kolb, J. A., Anderson, J. E., Studzinski, W. & Frusti, J. Refining economics of us gasoline: octane ratings and ethanol content. *Environmental science & technology* **48** (19), 11064–11071 (2014) .

[10] Alleman, T. L., McCormick, R. L. & Yanowitz, J. Properties of ethanol fuel blends made with natural gasoline. *Energy & Fuels* **29** (8), 5095–5102 (2015) .

[11] Monroe, E. *et al.* Discovery of novel octane hyperboosting phenomenon in prenol biofuel/gasoline blends. *Fuel* **239**, 1143–1148 (2019). URL https://www.sciencedirect.com/science/article/pii/S0016236118319288. https://doi.org/https://doi.org/10.1016/j.fuel.2018.11.046 .

[12] Vallinayagam, R. *et al.* Terpineol as a novel octane booster for extending the knock limit of gasoline. *Fuel* **187**, 9–15 (2017). URL https://www.sciencedirect.com/science/article/pii/S0016236116308924. https://doi.org/https://doi.org/10.1016/j.fuel.2016.09.034 .

[13] Christensen, E., Yanowitz, J., Ratcliff, M. & McCormick, R. L. Renewable oxygenate blending effects on gasoline properties. *Energy & Fuels* **25** (10), 4723–4733 (2011) .

[14] Tarazanov, S. *et al.* Assessment of the chemical stability of furfural derivatives and the mixtures as fuel components. *Fuel* **271**, 117594 (2020). URL https://www.sciencedirect.com/science/article/pii/S0016236120305895. https://doi.org/https://doi.org/10.1016/j.fuel.2020.117594 .

[15] Fayet, G. & Rotureau, P. How to use qspr-type approaches to predict properties in the context of green chemistry. *Biofuels, Bioproducts and Biorefining* **10** (6), 738–752 (2016) .

[16] Dahmen, M. & Marquardt, W. Model-based design of tailor-made biofuels. *Energy & Fuels* **30** (2), 1109–1134 (2016) .

[17] de Paulo, J. M., Barros, J. E. & Barbeira, P. J. A pls regression model using flame spectroscopy emission for determination of octane numbers in gasoline. *Fuel* **176**, 216–221 (2016). URL https://www.sciencedirect.com/science/article/pii/S001623611600154X. https://doi.org/https://doi.org/10.1016/j.fuel.2016.02.033 .

[18] Kelly, J. J., Barlow, C. H., Jinguji, T. M. & Callis, J. B. Prediction of gasoline octane numbers from near-infrared spectral features in the range 660-1215 nm. *Analytical Chemistry* **61** (4), 313–320 (1989) .

[19] van Leeuwen, J., Jonker, R. & Gill, R. Octane number prediction based on gas chromatographic analysis with non-linear regression techniques. *Chemometrics and Intelligent Laboratory Systems* **25** (2), 325–340 (1994). URL https://www.sciencedirect.com/science/article/pii/0169743994850518. https://doi.org/https://doi.org/10.1016/0169-7439(94)85051-8 .

[20] Abdul Jameel, A. G., Naser, N., Emwas, A.-H., Dooley, S. & Sarathy, S. M. Predicting fuel ignition quality using 1h nmr spectroscopy and multiple linear regression. *Energy & Fuels* **30** (11), 9819–9835 (2016) .

[21] Abdul Jameel, A. G., Van Oudenhoven, V., Emwas, A.-H. & Sarathy, S. M. Predicting octane number using nuclear magnetic resonance spectroscopy and artificial neural networks. *Energy & fuels* **32** (5), 6309–6329 (2018) .

[22] Naser, N., Yang, S. Y., Kalghatgi, G. & Chung, S. H. Relating the octane numbers of fuels to ignition delay times measured in an ignition quality tester (iqt). *Fuel* **187**, 117–127 (2017) .

[23] Pasadakis, N., Gaganis, V. & Foteinopoulos, C. Octane number prediction for gasoline blends. *Fuel Processing Technology* **87** (6), 505–509 (2006). URL https://www.sciencedirect.com/science/article/pii/S0378382006000051. https://doi.org/https://doi.org/10.1016/j.fuproc.2005.11.006 .

[24] Li, R., Herreros, J. M., Tsolakis, A. & Yang, W. Machine learning-quantitative structure property relationship (ml-qspr) method for fuel physicochemical properties prediction of multiple fuel types. *Fuel* **304**, 121437 (2021). URL https://www.sciencedirect.com/science/article/pii/S0016236121013168. https://doi.org/https://doi.org/10.1016/j.fuel.2021.121437 .

[25] Gao, Z., Cheng, X., Ren, F., Zhu, L. & Huang, Z. Compositional effects on sooting tendencies of diesel surrogate fuels with four components. *Energy & Fuels* **34** (7), 8796–8807 (2020) .

[26] McEnally, C. S. & Pfefferle, L. D. Sooting tendencies of oxygenated hydrocarbons in laboratory-scale flames. *Environmental science & technology* **45** (6), 2498–2503 (2011) .

[27] Das, D. D., McEnally, C. S. & Pfefferle, L. D. Sooting tendencies of unsaturated esters in nonpremixed flames. *Combustion and Flame* **162** (4), 1489–1497 (2015). URL https://www.sciencedirect.com/science/article/pii/S001021801400354X. https://doi.org/https://doi.org/10.1016/j.combustflame.2014.11.012 .

[28] Das, D. D. *et al.* Sooting tendencies of diesel fuels, jet fuels, and their surrogates in diffusion flames. *Fuel* **197**, 445–458 (2017). URL https://www.sciencedirect.com/science/article/pii/S0016236117301163. https://doi.org/https://doi.org/10.1016/j.fuel.2017.01.099 .

[29] McEnally, C. S. & Pfefferle, L. D. Improved sooting tendency measurements for aromatic hydrocarbons and their implications for naphthalene formation pathways. *Combustion and Flame* **148** (4), 210–222 (2007) .

[30] McEnally, C. S. & Pfefferle, L. D. Sooting tendencies of nonvolatile aromatic hydrocarbons. *Proceedings of the Combustion Institute* **32** (1), 673–679 (2009) .

[31] Kashif, M., Bonnety, J., Matynia, A., Da Costa, P. & Legros, G. Sooting propensities of some gasoline surrogate fuels: Combined effects of fuel blending and air vitiation. *Combustion and Flame*

162 (5), 1840–1847 (2015). URL https://www.sciencedirect.com/science/article/pii/S001021801400399X. https://doi.org/https://doi.org/10.1016/j.combustflame.2014.12.005 .

[32] McEnally, C. S. *et al.* Sooting tendencies of co-optima test gasolines and their surrogates. *Proceedings of the Combustion Institute* **37** (1), 961–968 (2019). URL https://www.sciencedirect.com/science/article/pii/S1540748918300725. https://doi.org/https://doi.org/10.1016/j.proci.2018.05.071 .
