# Supplementary material for: Artificial intelligence-driven design of fuel mixtures
Source: Commun Chem. 2022 Sep 16;5:111. doi: 10.1038/s42004-022-00722-3 (PMC9814251; doi:10.1038/s42004-022-00722-3)
Supplement: Supplementary file 2 — Description of Additional Supplementary Files.docx [file 42004_2022_722_MOESM2_ESM.docx]

Description of Additional Supplementary Files

**File name:** Supplementary Data 1

**Description: Calculated Jaccard–Tanimoto similarity score for five candidate mixtures. The scores were calculated for component pairs based on RDKit fingerprints.**

**File name:** Supplementary Data 2

**Description: Curated database for RON, MON and YSI experimental measurements for pure components. Training and test datasets for pure components used for model training and testing.**

**File name:** Supplementary Data 3

**Description: Curated database for RON, MON and YSI experimental measurements for mixtures. Training and test datasets for mixtures used for model training and testing.**
